# circRNA N6-methyladenosine methylation in preeclampsia and the potential role of N6-methyladenosine-modified circPAPPA2 in trophoblast invasion

**DOI:** 10.1038/s41598-021-03662-5

**Published:** 2021-12-21

**Authors:** Yonggang Zhang, Hongling Yang, Yan Long, Yipeng Zhang, Ronggui Chen, Junzhu Shi, Jiying Chen

**Affiliations:** 1 Department of Clinical Laboratory, Shenzhen Longhua District Central Hospital, Guangdong Medical University, No. 187 Guanlan Avenue, Shenzhen, 518110 Guangdong China; 2 Department of Clinical Laboratory, Guangzhou Women and Children’s Medical Centre, Guangzhou Medical University, Guangzhou, 510623 Guangdong China; 3 Department of Obstetrics and Gynecology, Shenzhen Longhua District Central Hospital, Guangdong Medical University, Shenzhen, 518110 Guangdong China

**Keywords:** Diseases, Molecular medicine, Pathogenesis

## Abstract

Here, we performed N6-methyladenosine (m6A) RNA sequencing to determine the circRNA m6A methylation changes in the placentas during the pathogenesis of preeclampsia (PE). We verified the expression of the circRNA circPAPPA2 using quantitative reverse transcription-PCR. An invasion assay was carried out to identify the role of circPAPPA2 in the development of PE. Mechanistically, we investigated the cause of the altered m6A modification of circPAPPA2 through overexpression and knockdown cell experiments, RNA immunoprecipitation, fluorescence in situ hybridization and RNA stability experiments. We found that increases in m6A-modified circRNAs are prevalent in PE placentas and that the main changes in methylation occur in the 3’UTR and near the start codon, implicating the involvement of these changes in PE development. We also found that the levels of circPAPPA2 are decreased but that m6A modification is augmented. Furthermore, we discovered that methyltransferase‑like 14 (METTL14) increases the level of circPAPPA2 m6A methylation and that insulin-like growth factor 2 mRNA-binding protein 3 (IGF2BP3) maintains circPAPPA2 stability. Decreases in IGF2BP3 levels lead to declines in circPAPPA2 levels. In summary, we provide a new vision and strategy for the study of PE pathology and report that placental circRNA m6A modification appears to be an important regulatory mechanism.

## Introduction

Preeclampsia (PE) is defined by elevated blood pressure and proteinuria or other organ damage that begins or is first recognized after 20 weeks of pregnancy^1^. It is one of the most common pregnancy-related disorder syndromes, with an incidence of approximately 3% to 8% worldwide^2,3^. PE can be injurious to the health of both the pregnant woman and the foetus. Furthermore, PE is associated with a higher and longer-term risk of cardiovascular disease development in both individuals^4^. Nevertheless, because the mechanism of PE pathogenesis has not yet been completely elucidated, effective means for the prevention or treatment of PE are not yet available.

As the placenta is closely associated with PE, it has become a pivotal target in the study of PE pathogenic processes^5^. It is generally accepted in the fields of obstetrics and gynaecology that PE occurs and develops as a consequence of long-term functional dislocation and molecular abnormalities of the placenta^5,6^. However, the molecular pathways and precise molecular mechanisms need to be further investigated.

N6-methyladenosine (m6A) is a newly identified major epigenetic modification of RNA. m6A, which was initially identified as an mRNA modification in mammals, is regarded as the most universal modification of mRNAs and non-coding RNAs in eukaryotes^7,8^. The latest research shows that reversible m6A modification can regulate RNA structure, stability, expression, and posttranslational modification by mediating various writer, eraser and reader proteins (such as methyltransferase‑like 3 (METTL3), fat mass- and obesity-associated protein (FTO) and YTH domain-containing family protein 2 (YTHDC2)) in all physiological and pathological situations^9,10^. The levels of m6A vary across different tissues^11^, and regulation of placental RNA m6A expression is unique.

Circular RNA (circRNA) is a new kind of noncoding RNA composed of a single-stranded loop that is formed mainly from direct back-splicing of pre-mRNA. CircRNAs act as miRNA sponges, regulate target genes or proteins and encode protein functions in the course of many diseases^12^, indicating that circRNAs may play a key role in the progression of diseases^13^. Previously, we reported a large number of dysregulated circRNAs in PE placentas^14^. Recent studies have also suggested that circRNAs may be important in PE^15^.

CircRNAs containing m6A modifications play important roles in many biological processes, including processes related to cancers and immune diseases^16^. Many studies have also shown that m6A is closely related to cardiovascular diseases, including hypertension^17^. Despite the publication of several articles on the relationship between m6A and hypertension disorders in pregnancy (including PE)^18,19^, there have been no reports on the correlation between circRNA m6A and PE. In this study, we examined the relationship between PE and circRNA m6A for the first time and sought to elucidate the mechanism of circPAPPA2 m6A modification in PE.

## Methods

### Patients and tissue samples

An authorization certificate was acquired from the local medical ethics committee (Medical Ethics Committee of Shenzhen Longhua District Central Hospital, approval number: 2018080). We obtained written consent from all participants. All procedures were performed in accordance with the guidelines set forth by the Declaration of Helsinki. PE and normal control pregnancy placenta samples were collected between February 2018 and May 2019 at Guangzhou Women and Children’s Medical Centre. Placental specimens were obtained by mixing four quadrants near the maternal side after delivery, and the samples were stored at -80 °C. PE was diagnosed by a senior professor of obstetrics and gynaecology according to the 2019 criteria of the American College of Obstetricians and Gynecologists (ACOG)^1^. The inclusion and exclusion criteria are shown in Supplementary Table S1.

### Methylated RNA immunoprecipitation (MeRIP) library preparation, RNA sequencing (RNA-Seq) and data analysis

The m6A RNA-Seq service was provided by Cloudseq Biotech, Inc. (Shanghai, China). Briefly, m6A RNA immunoprecipitation (IP) was performed with a GenSeq m6A-MeRIP Kit (GenSeq, Inc., China) following the manufacturer’s instructions. Both the input samples without IP and the m6A IP samples were used for RNA-Seq library generation with an NEBNext Ultra II Directional RNA Library Prep Kit (New England Biolabs, Inc., USA). The library quality was evaluated with a Bioanalyzer 2100 system (Agilent Technologies, Inc., USA). Library sequencing was performed using an Illumina NovaSeq instrument with 150-bp paired-end reads.

For data analysis, paired-end reads were obtained using an Illumina NovaSeq 6000 sequencer and quality-controlled according to the Q30 value. After 3’ adaptor trimming and low-quality read removal, Cutadapt software (v1.9.3) was used. First, clean reads of the input libraries were aligned to the reference genome (UCSC HG19) with STAR software, and circRNAs were identified with DCC software based on the STAR alignment results. The clean reads of all libraries were then aligned to the reference genome with HISAT2 software (v2.0.4). Methylated sites on RNA (peaks) were identified with MACS software. Differentially methylated sites were identified by diffReps. Peaks identified by both software programs with circRNA exon overlap were identified and selected with homemade scripts. Gene Ontology (GO) and pathway enrichment analyses were separately performed for differentially methylated protein-coding genes and the corresponding genes of differentially methylated circRNAs with DAVID 6.7 software. The links and version numbers of software in Table S2.

### Analysis of translation potential

The open reading frames (ORFs) of the circRNAs were identified with cORF software, and the internal ribosome entry sites (IRESs) of the circRNAs were predicted with IRESfinder software. CircRNAs that met the conditions (ORF length > 150 with an IRES and with start codon (startC) regions featuring different methylation sites) were selected. The inks and version numbers of software in Table S2.

### Verification of m6A levels (MeRIP-qRT-PCR)

Assays were carried out with a m6A-RNA methylation quantitative detection kit. Briefly, 3 μg of anti-m6A antibody (Abcam, USA, ab208577) was combined with protein A/G magnetic beads at 4 °C overnight. The antibody-bound beads and cell lysate were then incubated in IP buffer containing ribonuclease inhibitors and protease inhibitors. The bound RNAs were separated and detected by quantitative reverse transcription (qRT)-PCR.

### CircRNA fluorescence in situ hybridization (FISH)

A direct method for FISH was implemented based on the manufacturer's instructions to detect circPAPPA2 in TEV1 cells. Briefly, a circPAPPA2 probe was synthesized and labelled with the fluorescent substance Cy3 (RiboBio, Guangzhou, China). After fixation with 4% paraformaldehyde and treatment with 0.5% Triton X-100, cells were cultured with hybridization solution containing no probe for prehybridization to block nonspecificity. The hybridization reaction was carried out with a hybridizing solution containing a labelled probe (sequence provided in Supplementary Table S3) and cells overnight at 37 °C. Cellular DNA was counterstained with 4ʹ,6-diamidino-2-phenylindole (DAPI).

### qRT-PCR

Total RNA was extracted from cells or tissues with RNAiso Plus (TaKaRa, Dalian, China). After verifying the RNA quality, the RNA was reverse-transcribed using a GS II First Strand cDNA Synthesis Kit (Geneseed, Guangzhou, China). qRT-PCR was carried out with GS qPCR SYBR Green Master Mix and an ABI ViiA7 DX PCR instrument (Applied Biosystems, USA). Expression was quantified using the 2^−ΔΔCt^ method; glyceraldehyde-3-phosphate dehydrogenase (GAPDH) was used as a control. The primers used are shown in Supplementary Table S4.

### Immunohistochemistry (IHC)

IHC was carried out according to the manufacturer’s protocol. Antigen retrieval was performed with Tris–EDTA. Hydrogen peroxide was used as an endogenous peroxidase blocker. Placental sample slides were incubated with primary antibodies (anti-insulin like growth factor 2 mRNA binding protein 3 (IGF2BP3) antibody: ab231472, 1:100; anti-methyltransferase‑like 14 (METTL14) antibody: ab2223090, 1:200) and then with goat anti-rabbit secondary antibodies. Images were captured using a Leica microscope. A yellowish-brown colour was scored as positive (diaminobenzidine visualization).

### Cell transfection

Cells were obtained from the American Type Culture Collection (ATCC) (Shanghai, China) and YaJi Biological (Shanghai, China); custom siRNAs targeting circRNA were obtained from RiboBio (Guangzhou, China) and cloned into a lentiviral vector by Hanheng (Shanghai, China) (the siRNA target sequence is shown in Supplementary Table S5). Overexpressed circRNA was amplified by PCR and inserted into the lentiviral vector by Hanheng. The recombinant plasmids with specific sequences and supplementary packaging plasmids were transfected into 293 T cells with Lipofectamine 2000 transfection solution. After 48 h, the viral titres were measured, and then TEV1 cells (immortalized human extravillous trophoblast cells derived from a placenta that maintain the characteristics of extravillous trophoblasts) were infected with the virus. Finally, puromycin was used to select stable strains, and the cells with overexpression or knockdown were verified by qRT-PCR.

### Transwell invasion assay

A Transwell pore polycarbonate membrane insert (Corning, USA) was used for an invasion assay. Forty microlitres of diluted Matrigel (BD company, USA) was added to the Transwell chamber; 100 μL of serum-free medium (1 × 10^5^ cells per well) was then added to the upper chamber, and 600 μL of serum-free medium was added to the lower chamber. The Transwell was incubated in 5% CO_2_ at 37 °C for 24 h, and the cells in the lower chamber were fixed and stained.

### Analysis of circPAPPA2 stability

IGF2BP3-overexpressing TEV1 cells were plated in 6-well plates and reached 60% confluence after 24 h. The cells were treated with 5 μg/mL actinomycin D or DMSO for 0 h, 36 h or 72 h. The total RNA was treated with 3 U/μg RNase R and then subjected to qRT-PCR. The primers used are provided in Supplementary Table S4. The half-life of the circRNA was determined as described in previous publications^20,21^.

### RNA IP (RIP)

RIP was conducted using an EZ-Magna RIP Kit (Millipore, Germany) according to the manufacturer’s instructions. Magnetic beads labelled with an anti-m6A antibody (ab185912, Abcam), an anti-IGF2BP3 antibody (ab176685, Abcam) or anti-rabbit immunoglobulin G (IgG) were incubated at room temperature for 30 min. RIP IP buffer was added to each tube. Finally, the cell lysate supernatant was added to the magnetic bead-antibody complex, and the mixture was incubated overnight at 4 °C. The coprecipitated RNA was purified and quantified by qRT-PCR.

### Dual-luciferase reporter assay

Gene fragments of the wild-type circPAPPA2 3'-untranslated region (3'UTR) carrying wild-type m6A motifs and the mutant circPAPPA2 3'UTR carrying mutant motifs (in which m6A was replaced by T) were cloned downstream of the luciferase gene in the psiCHECK2 vector. Then, we cotransfected wild-type or mutant circPAPPA2-3'UTR psiCHECK2 reporter gene plasmids and the IGF2BP3 expression plasmid or control plasmid into HEK293T cells using Cellfectin II Reagent (Invitrogen). At 48 h after transfection, Renilla and firefly luciferase activity levels were assessed.

### Statistical analysis

Statistical analyses were performed using SPSS 24.0 (IBM) or GraphPad Prism 8.0. Differences between two groups were analysed with Student’s t-test or the Mann–Whitney U-test. *P* < 0.05 was considered to indicate statistical significance. The data are presented as the mean ± SD or as the number (percentage).

## Results

The characteristics of the participants are shown in Supplementary Table S6.

The experimental results are presented as two primary results and one secondary result. The primary results were the results of sequencing data analysis for circRNA m6A modification in PE and the results of functional/mechanistic experiments on the role of m6A-modified circPAPPA2 in PE. The secondary result was the preliminary prediction of circRNAs with translation potential.

### First primary result: sequencing data analysis for circRNA m6A modification in PE

#### m6A methylation levels of circRNAs are increased in PE placentas

**Figure 1:**
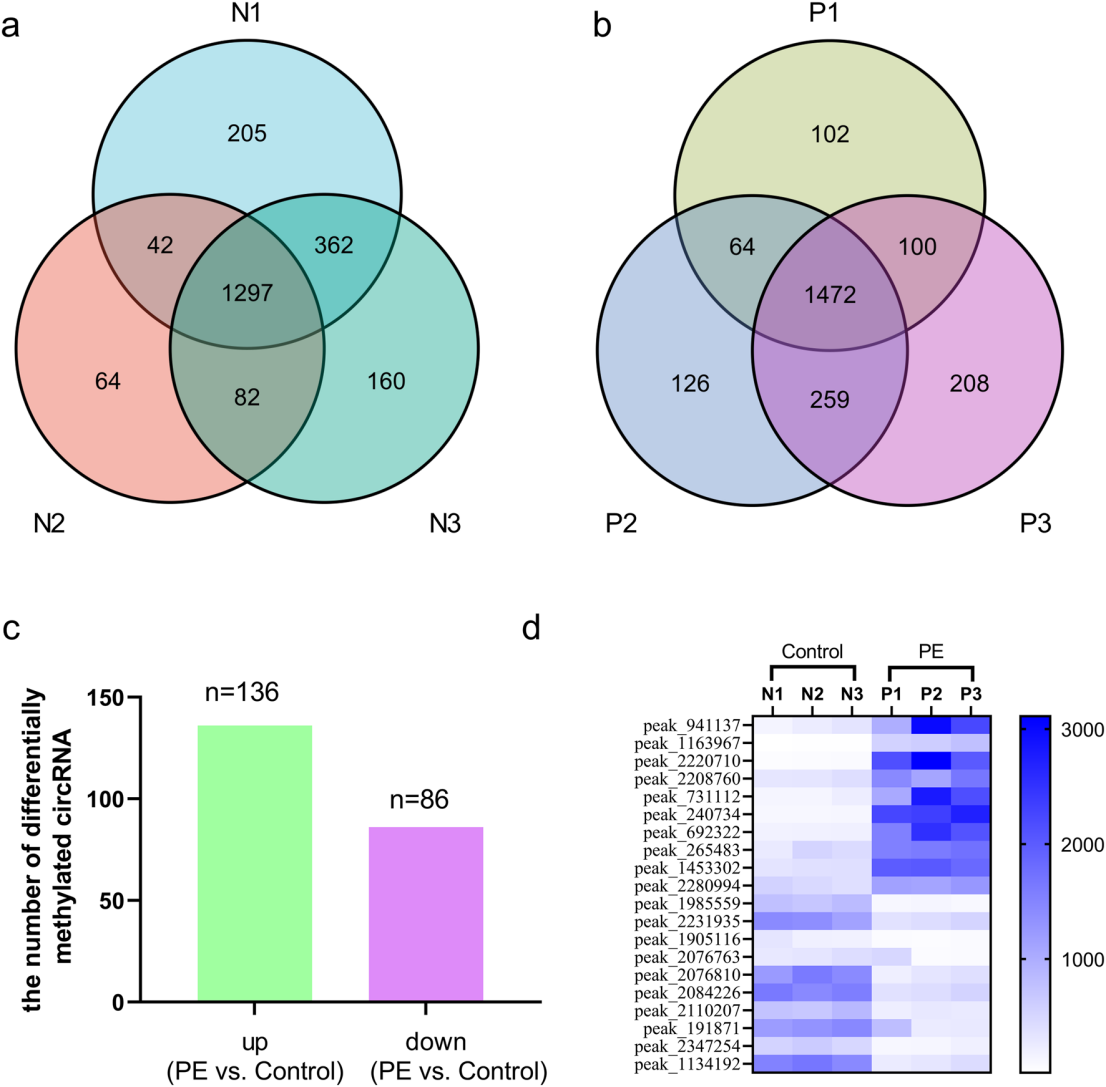
Results of m6A RNA sequencing data analysis with MACS and diffReps software. Venn diagram showing the overlap of m6A-modified sites on circRNAs among samples. The total number of m6A-modified sites on circRNAs in each group is shown (**a** P1–P3 represent three PE subgroups. **b** N1–N3 represent three control subgroups. MACS software was used). There were 136 upregulated m6A-methylated circRNAs and 86 downregulated m6A-methylated circRNAs in PE placentas compared to control placentas (**c** with diffReps Software, fold change > 2 and *P* < 10^–5^). Hierarchical clustering analysis of the top twenty differentially expressed methylation sites (**d**).

m6A RNA-Seq analysis with MACS software revealed that the three PE subgroups shared 1472 m6A-methylated sites of circRNAs (including different sites on the same circRNA, Fig. 1A), while the three control subgroups collectively showed 1297 m6A methylation sites of circRNAs (Fig. 1B). Therefore, overall m6A methylation levels on circRNA were higher in PE samples than in control samples.

#### Differentially methylated circRNAs in PE and control samples

m6A RNA-Seq analysis with diffReps software revealed that there were more upregulated m6A-methylated circRNAs (n = 136) than downregulated m6A-methylated circRNAs (n = 86) when the differentially expressed m6A peaks of circRNAs were compared between PE placentas and control placentas (Fig. 1C). This finding also indicates that m6A methylation has a certain role in PE. Hierarchical clustering analysis revealed the top twenty differentially expressed methylation sites in the 6 samples (blue indicates high expression and white indicates low expression in Fig. 1D).

#### m6A levels are elevated in the 3′UTRs of the corresponding circRNA transcripts in PE placenta specimens

**Figure 2:**
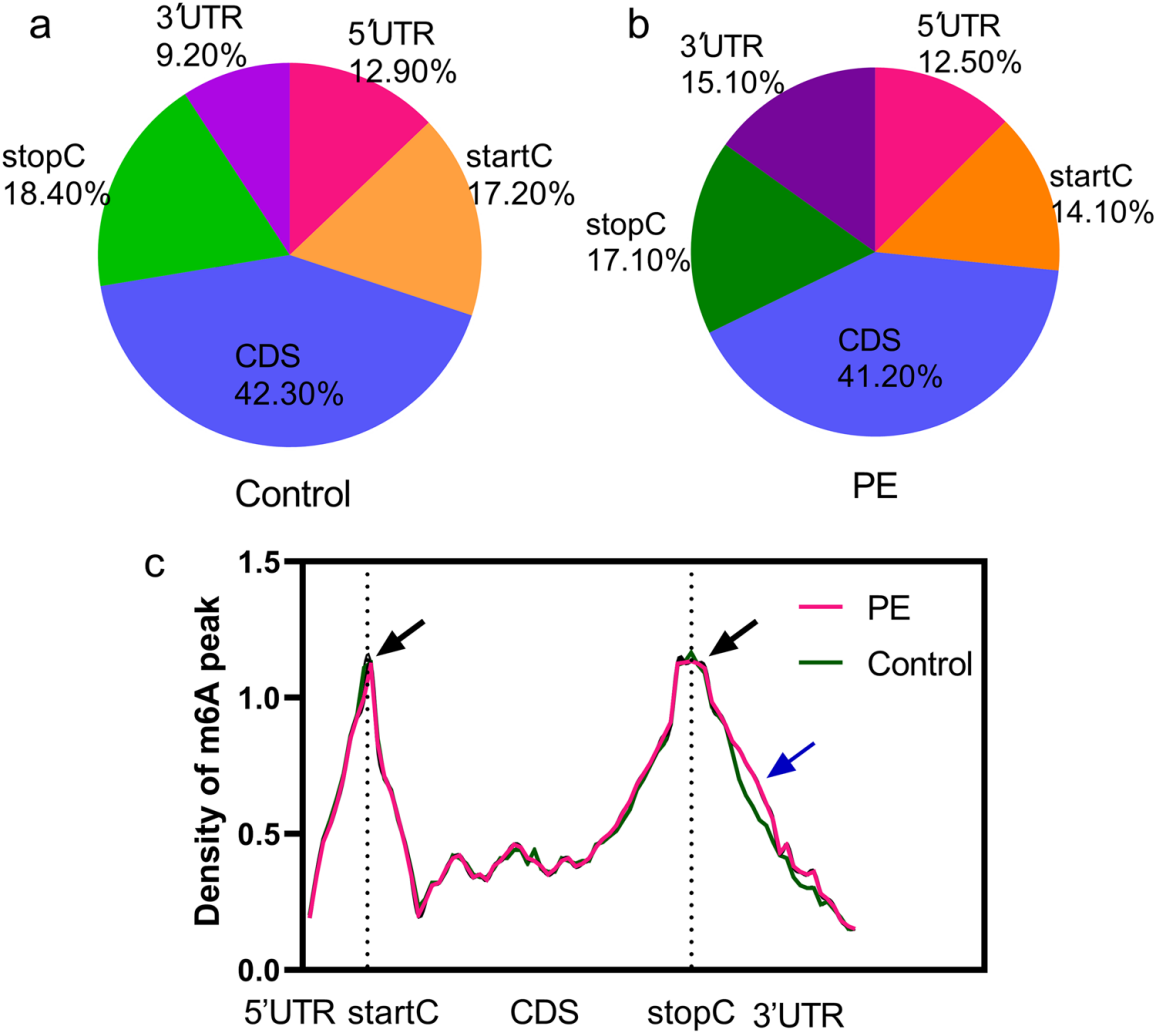
Results of m6A RNA sequencing data analysis with the HOMER or Python annotation tool. Proportions of m6A peaks distributed in the 5′-untranslated region (5′UTR), start codon (startC), coding DNA sequence (CDS), stop codon (stopC) and 3′-untranslated region (3′UTR) across the entire set of circRNA-corresponding transcripts in PE and control samples (**a**, **b**). Density distribution of m6A peaks across mRNA transcripts (**c**). m6A peaks were abundant near the startC or stop codon (stopC) (black arrow). We found that the density of m6A at the 3′UTR in PE placentas was higher than that in control placentas (blue arrow).

Further analysis of the m6A RNA-Seq results with the Homer or Python Annotation Tool indicated that the m6A peaks were mainly enriched in coding DNA sequence (CDS) regions (Fig. 2A,B). Nevertheless, the m6A levels in the 3′UTRs of circRNAs were higher in PE placenta specimens than in control specimens, whereas the m6A levels near the startCs were lower in PE placenta specimens than in control specimens (Fig. 2A,B). Moreover, m6A peaks were abundant near the startC or stop codon (stopC) (Fig. 2C, black arrow). We found that the density of m6A in the 3′UTR was higher in PE placentas than in control placentas (Fig. 2C, blue arrow).

#### GO and pathway analyses

**Figure 3:**
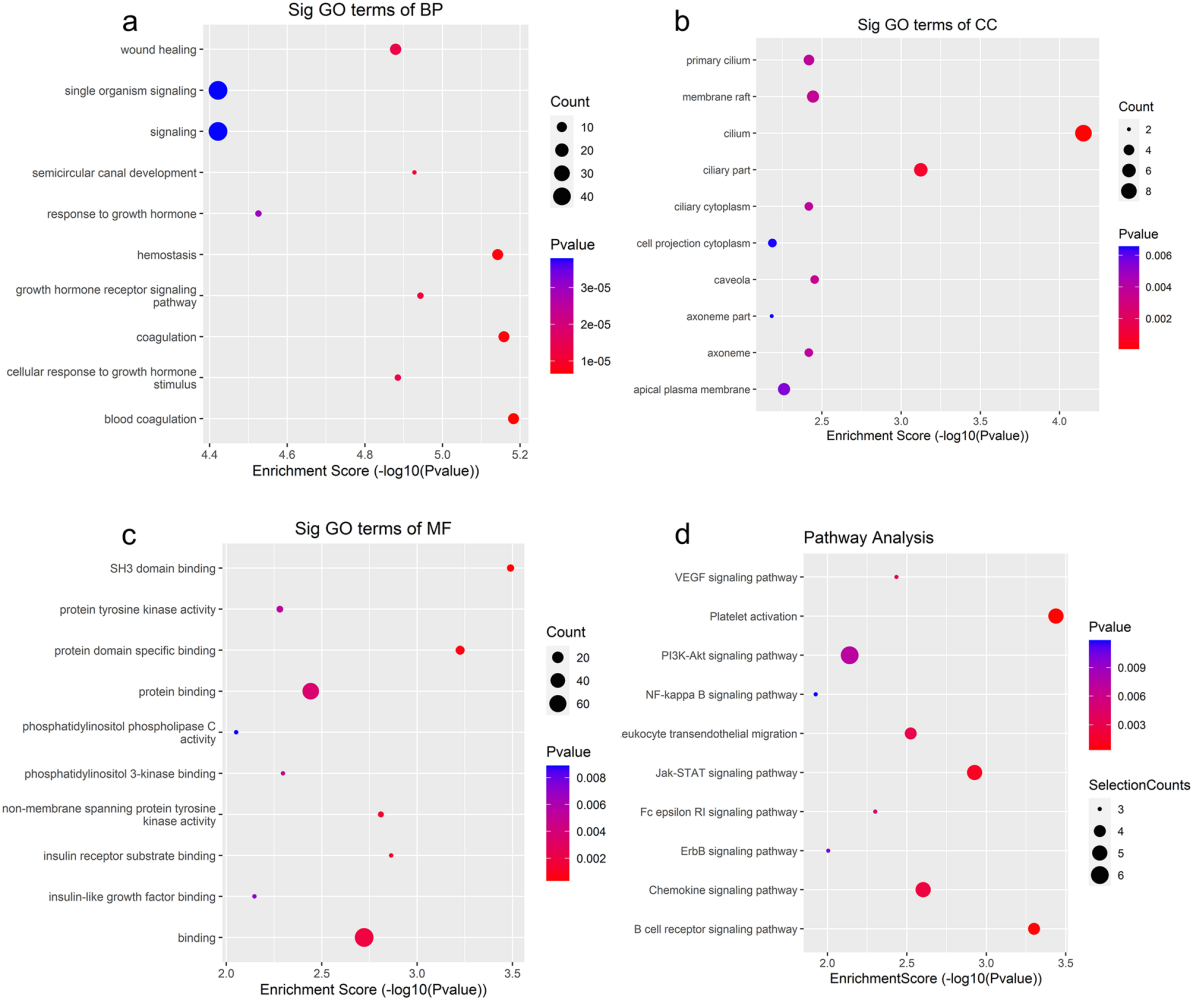
Results of m6A RNA sequencing data analysis with DAVID 6.7 software. Gene Ontology (GO) enrichment (**a**–**c**) and Kyoto Encyclopedia of Genes and Genomes (KEGG) signalling pathway (**d**) analyses for the host genes of upregulated m6A-methylated circRNAs. (BP: biological process; CC: cellular component; MF: molecular function).

Next, GO and pathway analysis were performed for the differentially expressed host genes. GO enrichment (in the biological process (BP), cellular component (CC), and molecular function (MF) categories) and Kyoto Encyclopedia of Genes and Genomes (KEGG) signalling pathway analyses of the host genes of the upregulated m6A-modified circRNAs (Fig. 3) revealed that platelet activation was the main signalling pathway. Blood coagulation was the main BP term, and SH3 domain binding was the major MF term.

#### The m6A modification levels of circPAPPA2 are enhanced in PE

**Figure 4:**
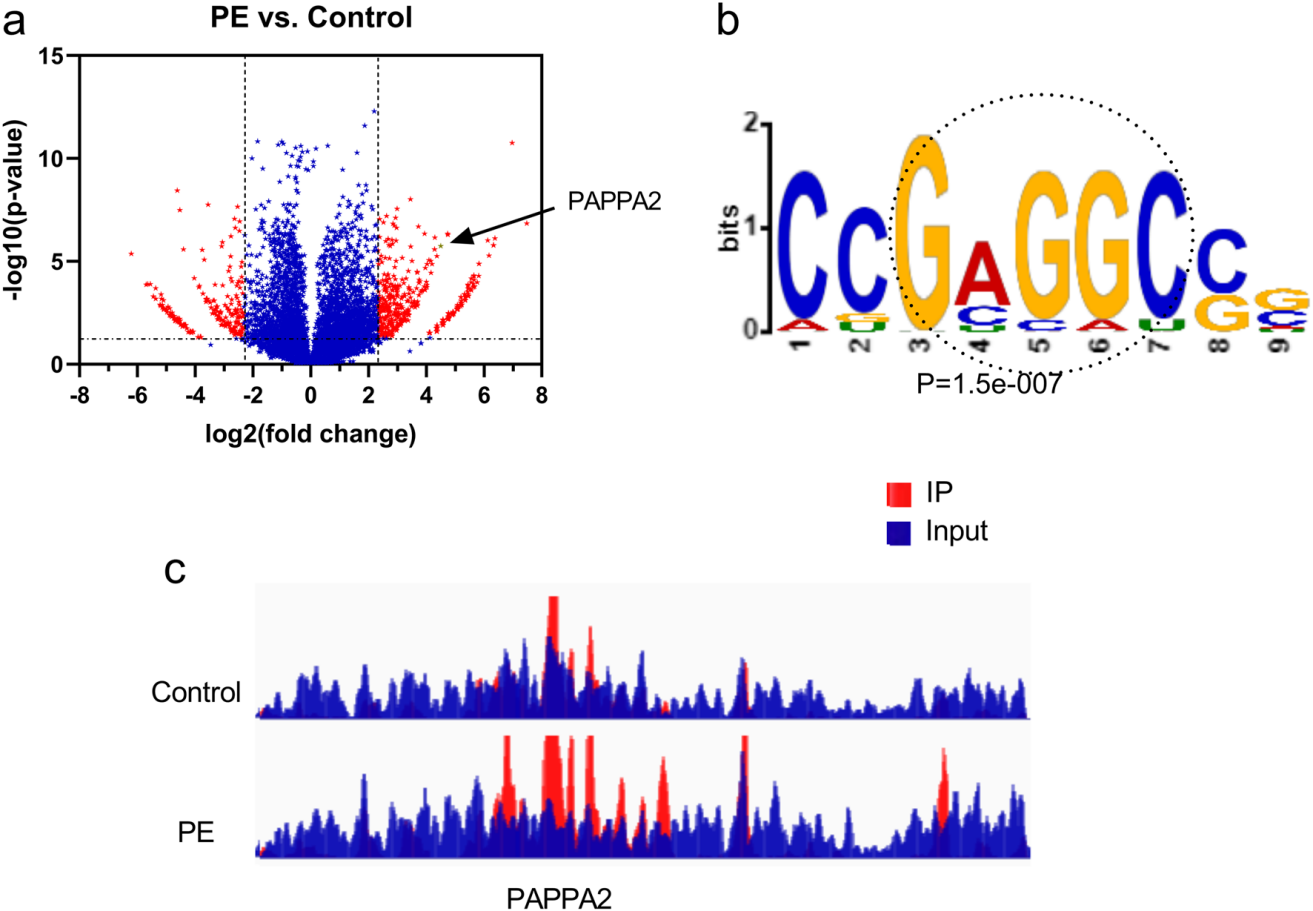
Volcano plots displaying distinct m6A-modified circRNAs and their statistical significance (fold change ≥ 5 and *P* < 0.05) (**a**). The main consensus motif (GAGGC) was identified in both the control and PE groups by sensitive, thorough, rapid enriched motif elicitation (STREME) of m6A sequencing data (**b**). m6A peaks were enriched in the CDS or 3′UTR of the PAPPA gene according to alignment visualization. The squares mark increases in m6A peaks in PE compared with control samples (IGV software, genome: Homo_sapiens_HG19) (**c**) (IP: m6A RNA immunoprecipitation).

Finally, through m6A RNA-Seq, we screened circRNAs with distinct m6A modification levels and determined their statistical significance using volcano plots (fold change ≥ 5 and *P* < 0.05). We found the m6A modification levels of circPAPPA2 to be increased in PE (Fig. 4A). Sensitive, thorough, rapid enriched motif elicitation (STREME) with m6A-Seq, we also observed that the main consensus motif (GAGGC) was present in the control and PE samples and that the circPAPPA2 transcript had the same motif (Fig. 4B). m6A peaks were enriched in the CDS or 3′UTR of the PAPPA2 gene according to alignment visualization, and marked increases in m6A peaks were observed in the PE group compared with the control group (Fig. 4C).

### Second primary result: Functions and mechanisms of m6A-modified circPAPPA2 in PE

#### circPAPPA2 expression is reduced in PE, and knockdown of circPAPPA2 suppresses trophoblast invasion

**Figure 5:**
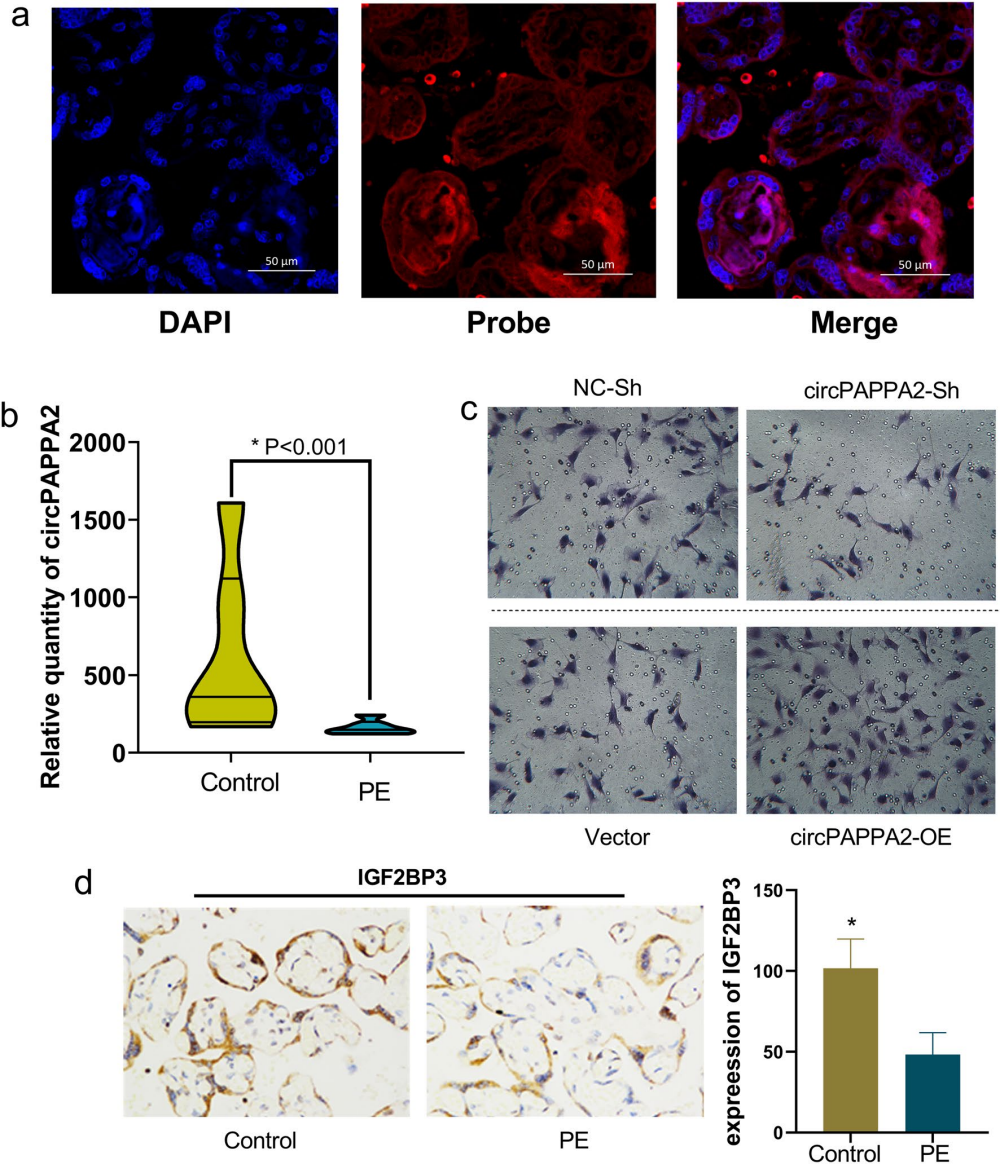
Fluorescent in situ hybridization assays showed that circPAPPA2 is located in the placenta (**a**). The expression of circPAPPA2 was increased in PE placentas compared to normal control placentas, as determined by RT-PCR (PE (n): control (n) = 6:6; *P* < 0.001) (**b**). Transwell invasion assays revealed that invasive capacity was significantly lower in the circPAPPA2 shRNA (circPAPPA2-sh) group than in the normal control shRNA (NC-Sh) group; the opposite result was obtained when circPAPPA2 was overexpressed (circPAPPA2-OE) (*P* < 0.05). Collectively, the results indicate that circPAPPA2 may promote trophoblast invasion (**c**). Insulin-like growth factor 2 mRNA binding protein 3 **(**IGF2BP3) was downregulated in PE placentas in the immunohistochemistry assay (*P < 0.05) (**d**).

The m6A level was verified with MeRIP-qRT-PCR (PE:control = 6:6), and the circPAPPA2 m6A levels were consistent with the m6A sequencing results. To study the potential role of circPAPPA2 in PE, we carried out circPAPPA2 localization and quantification and subsequently conducted an invasion experiment. We localized circPAPPA2 in the placenta via FISH (Fig. 5A) and found via qRT-PCR that circPAPPA2 was expressed at lower levels in PE placentas than in control placentas (Fig. 5B, PE:control = 6:6). We then carried out an invasion assay and demonstrated that knockdown of circPAPPA2 in TEV1 cells inhibited the invasion of these trophoblast cells but that the opposite result was obtained when circPAPPA2 was overexpressed (Fig. 5C). The assays indicated that downregulating the expression of circPAPPA2 inhibits trophoblast invasion.

#### Downregulation of IGF2BP3 reduces circPAPPA2 stability and leads to a decline in the circPAPPA2 level

**Figure 6:**
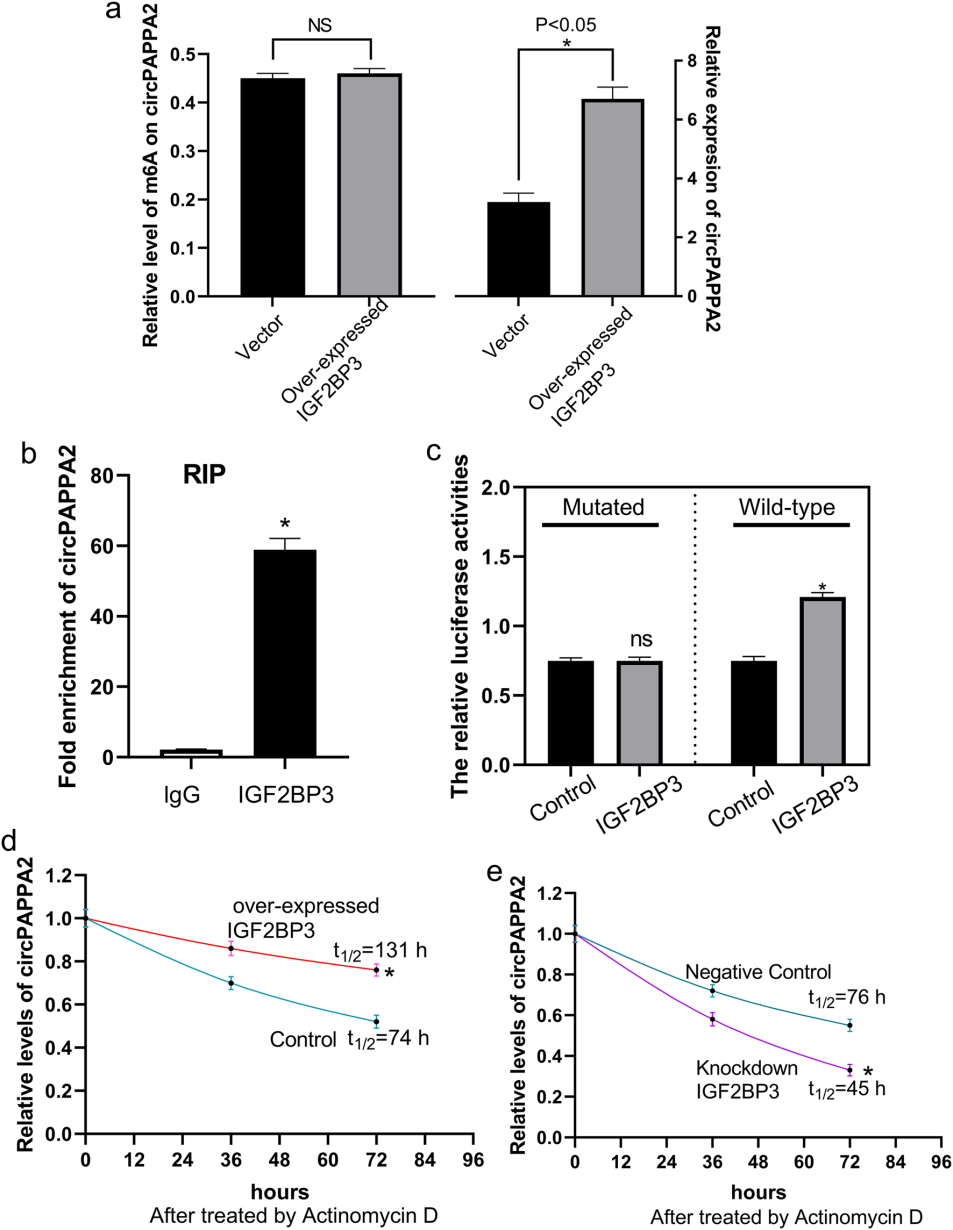
Through overexpression experiments, insulin-like growth factor 2 mRNA binding protein 3 **(**IGF2BP3) was found to increase circPAPPA2 expression but have no effect on the levels of m6A on circPAPPA2 (**a**). RNA immunoprecipitation (RIP) revealed that IGF2BP3 can bind to circPAPPA2 (**b**). Luciferase assay results showed that downregulation of IGF2BP3 can downregulate circPAPPA2 expression in an m6A-dependent manner (**c**). In an analysis of circPAPPA2 stability, after transcriptional inhibition using actinomycin D, overexpression of IGF2BP3 increased the circPAPPA2 half-life (**d**); knockdown of IGF2BP3 in TEV1 cells shortened the circPAPPA2 half-life (**e**) (all *P < 0.05).

To investigate the mechanism of low expression of circPAPPA2 in PE, the m6A reader protein IGF2BP3 was identified, and a series of mechanistic experiments were performed. IGF2BP3 was downregulated in PE placentas (Fig. 5D). When IGF2BP3 was overexpressed, the levels of m6A on circPAPPA2 did not differ significantly, but circPAPPA2 expression was increased (Fig. 6A). RIP with an anti-IGF2BP3 antibody showed higher fold enrichment of circPAPPA2 than the control in TEV1 cells (Fig. 6B), revealing that IGF2BP3 can bind to circPAPPA2. Furthermore, to evaluate the effects of IGF2BP3 binding, a dual luciferase reporter assay was performed with the 3’UTR of circPAPPA2. The results showed that IGF2BP3 overexpression significantly elevated luciferase expression activity in the wild-type 3’UTR group (Fig. 6C). However, luciferase activity was suppressed by point m6A site mutation (Fig. 6C), showing that a reduction in IGF2BP3 can decrease circPAPPA2 expression in an m6A-dependent manner. Thus, we analysed the effect of IGF2BP3 on the circPAPPA2 half-life by sampling IGF2BP3-overexpressing TEV1 cells at different time points after transcriptional inhibition using actinomycin D. As expected, overexpression of IGF2BP3 resulted in a longer circPAPPA2 half-life (Fig. 6D). In line with this finding, knockdown of IGF2BP3 in TEV1 cells shortened the circPAPPA2 half-life (Fig. 6E). In summary, IGF2BP3 is able to identify m6A modifications and bind to circPAPPA2, increasing its stability.

#### Upregulation of METTL14 increases circPAPPA2 m6A methylation

**Figure 7:**
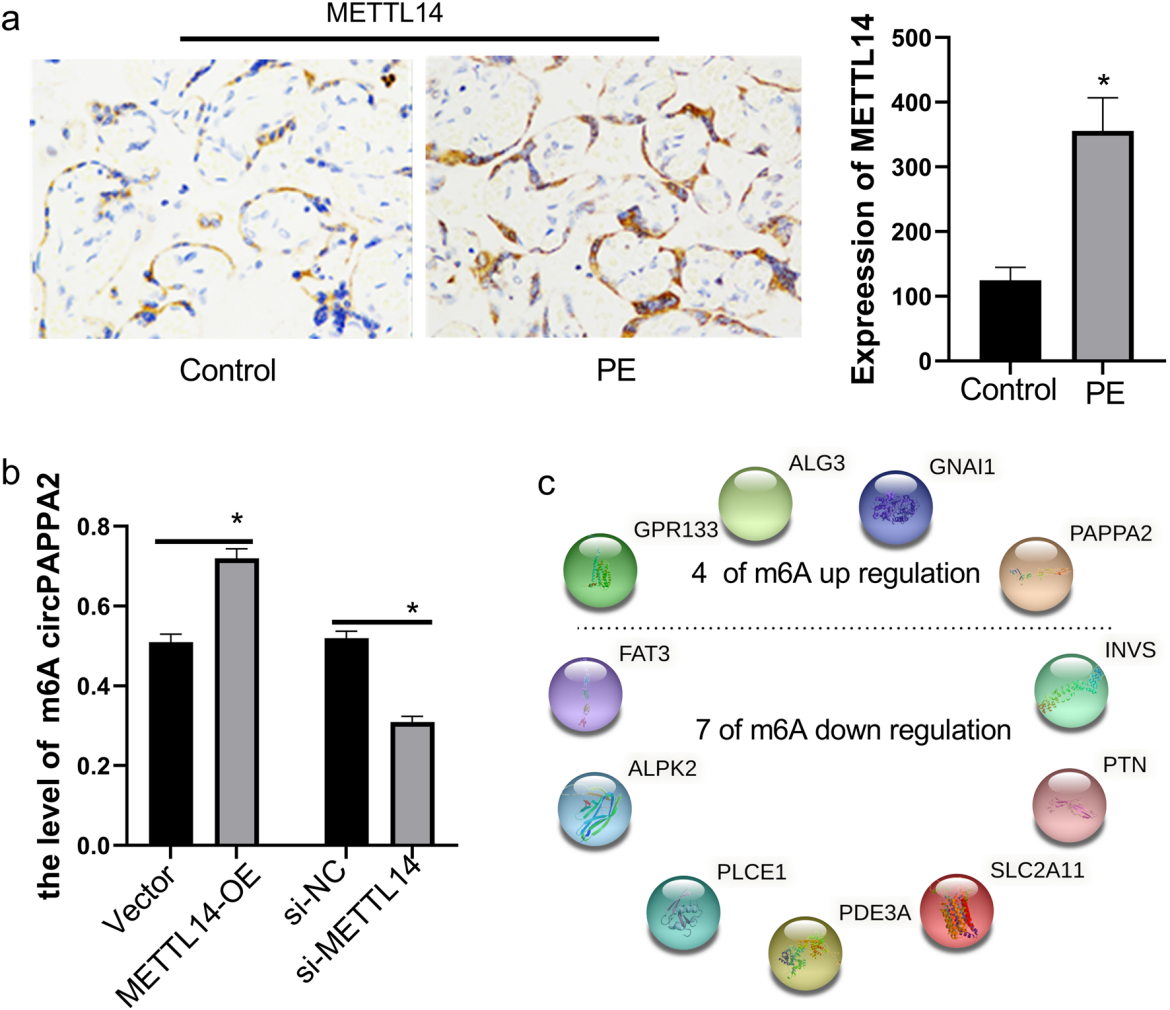
METTL14 showed increased expression in PE placentas in the immunohistochemistry assay (*P < 0.05) (**a**). When METTL14 was overexpressed in TEV1 cells, the circPAPPA2 m6A methylation level increased. With si-METTL14, the level of circPAPPA2 m6A methylation decreased (*P < 0.05) (**b**). There were 4 upregulated and 7 downregulated m6A-methylated circRNAs with the potential to encode proteins according to m6A RNA sequencing data analysis using cORF software and IRESfinder software (**c**). circRNAs were selected based on an ORF length > 150, the presence of an IRES, and the presence of start codon regions with different methylation sites (GPR133, ALG3, GNAI1, PAPPA2, PLCE1, INVS, SLC2A11, PDE3A, FAT3, PTN, and ALPK2 represent circGPR133, circALG3, circGNAI1, circPAPPA2, circPLCE1, circINVS, circSLC2A11, circPDE3A, circFAT3, circPTN, and circALPK2, respectively).

To explore the cause of the increased level of circPAPPA2 m6A methylation, m6A writer proteins were identified with overexpression and knockout experiments. In this study, we confirmed that the levels of the m6A writer protein METTL14 were increased in PE (Fig. 7A). When METTL14 was overexpressed in TEV1 cells, the level of circPAPPA2 m6A methylation also increased. In contrast, the level of circPAPPA2 m6A methylation in placental trophoblasts decreased with METTL14 siRNA (si-METTL14) treatment (Fig. 7B).

Functional and mechanistic experiments showed that the circRNA circPAPPA2 mediates the expression of m6A writer METTL14 and the m6A reader IGF2BP3 to increase self-methylation and maintain self-stability, inhibiting trophoblast invasion in PE.

### In addition to the two primary results, a secondary result was obtained: preliminary prediction of circRNAs with translation potential

#### Partially m6A-methylated circRNAs may encode proteins

Some circRNAs may be able to be translated into proteins through m6A modification. To identify potential circRNAs encoding proteins, a bioinformatics analysis was executed with cORF software and IRESfinder software. The results identified 4 upregulated and 7 downregulated m6A-methylated circRNAs that may have the potential to encode proteins, as shown in Fig. 7C. These circRNAs met several conditions: they had ORFs > 150 bases in length, they had IRESs, and they had startC regions with different methylation sites.

## Discussion

The placenta undoubtedly plays a role in PE pathogenesis. m6A modification is prevalent and functionally relevant in eukaryotes^8^, and the role of m6A modification has been well defined in mRNA. To date, there have been a few reports about mRNA m6A modification in PE placentas^18,19^, although the m6A modification profiles of circRNAs in PE have not yet been characterized. In posttranscriptional modification, widespread reverse splicing of pre-mRNA results in tens of thousands of circRNAs; however, the molecular functions of these circRNAs need to be further elucidated. Yun Yang et al. reported that m6A, the most abundant base modification of RNA, can promote the translation ability of circRNA, and a number of circRNAs have translation potential^22^.

To our knowledge, this study is the first to observe a correlation between circRNA m6A methylation and PE using m6A RNA-Seq. Integrated analysis of the data revealed that overall m6A methylation levels were increased in PE placentas. Further analysis suggested that the predominant changes in m6A levels in PE placental specimens occurred at the 3'UTR and near the startC. GO analysis and pathway analysis were conducted, and the results support previous studies on coagulation and the vascular endothelium in PE^23^. Moreover, the levels of circPAPPA2 m6A modification were enhanced in PE, but circPAPPA2 expression was reduced in PE, and decreased expression of circPAPPA2 attenuated trophoblast invasion. Ultimately, mechanistic analysis showed that increases in the levels of the m6A writer METTL14 augmented the levels of circPAPPA2 m6A methylation but that decreases in the levels of the m6A reader IGF2BP3 reduced circPAPPA2 stability, leading to declines in circPAPPA2 levels. Overall, we identified the effect of circPAPPA2 m6A modification on trophoblast invasion in PE. A decrease in the level of circPAPPA2 suppressed trophoblast invasion, and circPAPPA2 was downregulated via modulation of its stability through m6A methylation.

Recent studies have reported that IGF2BPs, members of a distinct m6A reader family, can identify specific m6A methylation modifications on the transcripts of target genes^24^. Unlike YTHDC, which can reduce RNA stability^25^, IGF2BPs promote the stability of target RNAs in an m6A-dependent manner, thus modulating RNA levels. It has been reported that METTL14 enhances methylation of RNA^26^, consistent with our research. According to previous reports, three basic elements endow circRNAs with a unique ability to encode proteins: IRESs, ORFs and m6A modifications. In this study, using m6A modification data, cORF software and IRESfinder software, we also found that 11 circRNAs (4 with increased m6A modification and 7 with decreased m6A modification) possessed translation potential. The finding that these circRNAs are likely translated into proteins needs to be further studied.

In conclusion, our findings implicate m6A on circRNAs in the development of PE, broadening the options for the management of PE. Furthermore, we demonstrate that the m6A reader IGF2BP3 is essential for the maintenance of circPAPPA2 stability in an m6A-dependent manner. Moreover, we report that several circRNAs (such as circPAPPA2) may have protein-coding potential in PE.

## Supplementary Information

Supplementary Tables.

## Data Availability

The raw data required cannot be shared at this time, as the data also form part of an ongoing study. However, the raw data will be available at the Gene Expression Omnibus (GEO). The processed data associated with the current study are available from the corresponding author upon reasonable request.
