# Supplementary material for: circRNA N6-methyladenosine methylation in preeclampsia and the potential role of N6-methyladenosine-modified circPAPPA2 in trophoblast invasion
Source: Sci Rep. 2021 Dec 21;11:24357. doi: 10.1038/s41598-021-03662-5 (PMC8692596; doi:10.1038/s41598-021-03662-5)
Supplement: Supplementary file 1 — Supplementary Tables. [file 41598_2021_3662_MOESM1_ESM.docx]

**Full title: circRNA N6-methyladenosine methylation in preeclampsia and the potential role of N6-methyladenosine-modifi****ed circPAPPA2 in trophoblast invasion**

Yonggang Zhang^1^**🖂**, Hongling Yang^2^, YanLong^2^, Yipeng Zhang^1^, Ronggui Chen^1^**🖂**, Junzhu Shi^1^, Jiying Chen^3^

1. Department of Clinical Laboratory, Shenzhen Longhua District Central Hospital, Guangdong Medical University, Shenzhen, Guangdong, 518110, China;

2. Department of Clinical Laboratory, Guangzhou Women and Children’s Medical Centre, Guangzhou Medical University, Guangzhou, Guangdong, 510623, China.

3 Department of Obstetrics and Gynecology, Shenzhen Longhua District Central Hospital, Guangdong Medical University, Shenzhen, Guangdong, 518110, China;

**Table S1. Inclusive and exclusive conditions**

| **Inclusive conditions** | **exclusive conditions** |  |
| --- | --- | --- |
| pregnancy women with preeclampsia, and normal healthy pregnancy women with normal blood pressure, without proteinuria and any medical or pregnancy complications. the cases were matched with normal control in a 1:1 ratio for age, gestational week and body mass index. | Trophoblastic disease, multiple pregnancy, in vitro fertilization treatment, gynecological disease, individual taking medicine (especially aspirin, antibiotics), alcohol intake, liver and kidney diseases, autoimmune diseases etc. |  |
|  |  |  |

**Table S2.** **Links and version numbers of software in data analysis of RNA sequencing**

| Software | Version | Software_link |
| --- | --- | --- |
| Cutadapt | 1.9.3 | https://github.com/marcelm/cutadapt/ |
| STAR | 2.6.1a | https://github.com/alexdobin/STAR |
| DCC | 0.4.7 | https://github.com/dieterich-lab/DCC |
| HISAT2 | 2.0.4 | http://ccb.jhu.edu/software/hisat2/index.shtml |
| MACS | 1.4.2 | https://pypi.org/project/MACS/1.4.2/ |
| DAVID | v6.7 | https://david.ncifcrf.gov/ |
| cORF | - | https://github.com/kadenerlab/cORF_pipeline |
| IGV | 2.8.3 | https://software.broadinstitute.org/software/igv/download |
| diffReps | 1.55.3 | https://metacpan.org/dist/diffReps |
| IRESfinder | 1.1.0 | https://github.com/xiaofengsong/IRESfinder |

**Table S3. Probe Sequence for FISH.**

|  | **Probe Sequence** |
| --- | --- |
| **circPAPPA2** | ATTCGTAGACATAGCCTACTTGGATGTTAATGAGCTGAAGAACATT |

**Table S4. Primers Sequence for PCR or RT-PCR**

|  | **GAPDH or primers Sequence** |
| --- | --- |
| **GAPDH-F:** | 5'-AGAAGGCTGGGGCTCATTTG-3' |
| **GAPDH-R:** | 5'-GCAGGAGGCATTGCTGATGAT-3' |
| **circ_PAPPA2-F:** | 5'-AGGAGGAAACAAAGGGTGAATG-3' |
| **circ_PAPPA2-R:** | 5'-CAGCAGCACTGAGGTGGTATGG-3' |

**Table S5. shRNA against Human circPAPPA2**

| **circRNA name** | **shRNA Target Sequence** |
| --- | --- |
|  | #2 GCAAGGGGCATGACTCTCTCT |
|  |  |

**Table S6. Clinical characteristics for Mothers and newborns in PE and control group.**

|  | **PE (n=6)** | **Control (n=6)** | ***P*** |
| --- | --- | --- | --- |
|  | **Mean ± SD or N (%)** | **Mean ± SD or N (%)** |  |
| Gestational week (week) | 39±1.73 | 38.33±0.58 | 0.065 |
| **Demographic characteristics** |  |  |  |
| The Han nationality n (%) | 3(100) | 3(100) | —— |
| Maternal age (year) | 27.67±3.06 | 27.67±2.31 | 0.653 |
| BMI(kg/m^2^) | 25.07±1.67 | 24.6±3.20 | 0.424 |
| **Maternal adverse outcomes** |  |  |  |
| Caesarean section n (%) | 0 (0%) | 0 (0%) | —— |
| **Neonatal outcomes** |  |  |  |
| newborn weight | 2.88±0.18 | 3.27±0.13 | 0.632 |

BMI:body mass index
